# Immune profiling in human breast cancer is predictive of 5- and 10-year survival.

**DOI:** 10.1186/2051-1426-3-S2-P131

**Published:** 2015-11-04

**Authors:** Brendon Coventry, Michael J Weightman, John Bradley, John M Skinner

**Affiliations:** 1 Discipline of Surgery, University of Adelaide & Breast, Endocrine & Surgical Oncology Unit, Royal Adelaide Hospital, Australia; 2 Department of Clinical Immunology, Flinders University, Flinders Medical Centre, Adelaide, Australia; 3 Pathology, Flinders University, Flinders Medical Centre, Adelaide, Australia

## Background

Immunotherapy for breast cancer is now being seriously considered, despite past beliefs that this cancer type was non-immunogenic. Immune profiling has been variably associated with outcome, but standard techniques have greatly limited interpretation. These studies examine immune profiling of breast cancer patients using high-sensitivity methods in relation to clinical outcome.

## Methods

High-sensitivity detection and analysis methods were used to determine Immune Profiles of female human breast cancer in Tumour Infiltrating Leukocytes (TIL) in longitudinal cohort comparative outcome studies.

## Results

Immune profiles showed predominantly CD3, CD4, CD45RO TIL with low/absent IL-2a receptor expression. However, these were significantly correlated to 5- and 10-year survival times.

## Conclusions

Immune profiling of the TIL infiltrate in human breast carcinoma using high-sensitivity detection and analysis techniques showed predominance of CD3 cells, being ab-TCR, CD4 T cells of mainly memory phenotype. Importantly, these findings were strongly predictive of 5- and 10-year survival. This indicates the real possibility that TIL infiltration in breast cancer might be open to immunological manipulation therapeutically to improve clinical outcome.

